# Graphene Multiple Fano Resonances Based on Asymmetric Hybrid Metamaterial

**DOI:** 10.3390/nano10122408

**Published:** 2020-12-02

**Authors:** Zhendong Yan, Zhixing Zhang, Wei Du, Wenjuan Wu, Taoping Hu, Zi Yu, Ping Gu, Jing Chen, Chaojun Tang

**Affiliations:** 1 College of Science, Nanjing Forestry University, Nanjing 210037, China; zdyan@njfu.edu.cn (Z.Y.); zhangzhixing1220@gmail.com (Z.Z.); wjwu@njfu.edu.cn (W.W.); fox_tphu@sina.com (T.H.); ziyu_njfu@163.com (Z.Y.); 2 College of Physics Science and Technology, Yangzhou University, Yangzhou 225002, China; wdu@yzu.edu.cn; 3 College of Electronic and Optical Engineering & College of Microelectronics, Nanjing University of Posts and Telecommunications, Nanjing 210023, China; guping@njupt.edu.cn (P.G.); jchen@njupt.edu.cn (J.C.); 4 College of Science, Zhejiang University of Technology, Hangzhou 310023, China

**Keywords:** graphene, multiple Fano resonances, dark mode, metamaterial

## Abstract

We theoretically investigate multiple Fano resonances in an asymmetric hybrid graphene–metal metamaterial. The multiple Fano resonances emerge from the coupling of the plasmonic narrow bonding and antibonding modes supported by an in-plane graphene nanoribbon dimer with the broad magnetic resonance mode supported by a gold split-ring resonator. It is found that the Fano resonant mode with its corresponding dark mode of the antibonding mode in the in-plane graphene nanoribbon dimer is only achieved by structural symmetry breaking. The multiple Fano resonances can be tailored by tuning the structural parameters and Fermi levels. Active control of the multiple Fano resonances enables the proposed metamaterial to be widely applied in optoelectronic devices such as tunable sensors, switches, and filters.

## 1. Introduction

Optical Fano resonance (FR) is a typical destructive interference effect resulting from the strong coupling between spectrally overlapping narrow discrete and broad excitation modes, which shows a sharp and asymmetric line shape and produces a large electromagnetic field congregation [1,2]. In recent years, single FRs have been realized from visible to far-infrared in different kinds of metamaterials and metallic nanostructures, and many efforts have been devoted to figure out their highly efficient generations and tunabilities [3,4,5,6,7,8,9,10]. Moreover, multiple FRs in different metallic nanostructures have also gained much attention [11,12,13,14,15]. Compared to a single FR, multiple FRs can find even more flexible and diversified applications in integrated photonic devices, such as plasmon rulers [16], solar cells [17], multicolor nonlinear processes [18,19], and wavelength division multiplexers [20]. Multiple FRs can be realized by constructing the meta-molecules for near-field coupling between multiple bright and dark meta-atoms [21,22]. Furthermore, the structural symmetry breaking enables the generation of new narrow dark modes as well as multiple FRs, which has been reported in various nanostructures [2,4,11,23,24,25,26,27,28].

Monolayer graphene has become a new focus of research in active control of FRs effects thanks to its exceptional electrical and optical properties, such as its ultrahigh electron mobility and tunable conductivity for flexible plasmon excitations [20,21,22]. Supporting both high electric field confinement and low loss at terahertz regions, graphene plasmon modes possess much narrower linewidths compared to those of metals. Over recent years, sorts of hybrid graphene and metal metamaterials have been proposed by taking advantage of the narrow plasmon lineshape of graphene interacted with the broad optical mode of metallic resonators, showing a strong single FR effect [23,24,25,29,30]. However, there is seldom research about multiple FRs of the hybrid graphene–metal system [31], which is highly desirable. For example, Zhang et al. and Guo et al. reported that different higher-order plasmon modes of patterned graphene acting as narrow modes interact with a broad dipolar mode to generate different higher-order FRs in pure graphene or hybrid graphene–metal metamaterials [27,32]. On the other hand, plasmon hybridization among neighboring graphene structures is an effective way to generate new narrow hybridized plasmon modes through optical near-field coupling [33,34,35]. A previous effort was focused on achieving the narrow electric dipole symmetric and antisymmetric modes by plasmon hybridization in a stacked graphene nanoribbon pair, and the formation mechanism of double FRs in a symmetrical structure of a graphene–metal metamaterial [36]. It is noteworthy that the two narrow electric dipole symmetric and antisymmetric modes are still the plasmonic bright modes, which can be directly excited by light under normal incidence. However, the plasmonic dark mode of graphene, as well as its related single FR or multiple FRs, has not yet been reported, which is quite in demand for developing graphene-based optoelectronic devices.

In this work, we demonstrate that a properly designed graphene–metal system with decreased structural symmetry can exhibit plasmonic multiple FRs with narrow linewidths. We adopt a commonly used hybrid structure composed of a gold split-ring resonator (SRR) and an in-plane graphene nanoribbon dimer (GNRD), where the magnetic dipole resonance of the gold SRR acts as a broad mode and the plasmonic hybridized in-phase and out-of-phase bonding resonant modes as well as the antibonding resonant mode excited in the GNRD act as the three narrow modes. Interestingly, it is found that one Fano resonance with its corresponding narrow dark mode being the antibonding modes of the GNRD is only excited via the introduction of structural symmetry breaking in the graphene–metal metamaterial. By decreasing the vertical coupling distance between the GNRD and SRR, extra Fano transmission peaks can be further obtained. The active control of the multiple transparency windows is demonstrated by tuning graphene’s Fermi energy and electronic mobility of the GNRD. The proposed hybrid metamaterial can be realized without changing the structure, suggesting potential applications such as tunable sensors, switchers, and filters.

## 2. Methods

**Figure 1:**
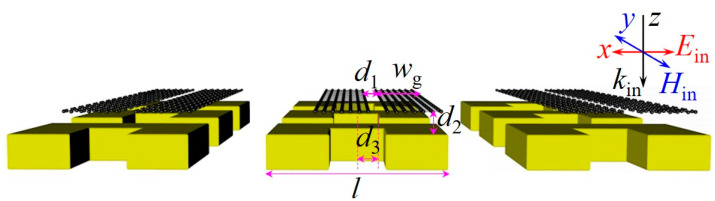
Schematic of the hybrid graphene–metal metamaterial.

Figure 1 schematically shows the array of the graphene–metal metamaterial, which consists of the GNRD along the *y* direction and a gold SRR. The electric field *E*_in_, magnetic field *H*_in_, and wave vector *K*_in_ of the incident light are along the *x*, *y*, and *z* directions, respectively. The gap size *d*_1_ between the two in-plane graphene nanoribbons, the vertical distance *d*_2_ between the GNRD and the gold SRR, and the offset *d*_3_ between the geometrical centers of the constituent hybrid metamaterial in the *x* axis are also shown. The arm length *l*, arm width *w*, and thickness *t* of the SRR are fixed at 3 μm, 0.75 μm, and 30 nm, respectively. The width *w*_g_ of the graphene nanoribbon (GNR) is fixed at 0.65 μm, while the length of the GNR is infinite along the *y* direction. The periodicity *P* along both *x* and *y* directions is set as 6 μm. The surrounding media are assumed to be air for simplicity (*ε*_d_ = 1).

The dielectric properties of gold were calculated by using the Drude model [37] with a plasma frequency of *ω*_p_ = 1.37 × 10^16^ s^−1^ and a collision frequency of *ω*_c_ = 4.08 ×10^13^ s^−1^. The thickness (*t*_g_) of monolayer graphene was set as 1 nm. The graphene surface conductivity (*σ*_g_) including the intraband and interband transition contribution was calculated by the local limit of random-phase approximation [21]. The dielectric constant of graphene can be further obtained by $\varepsilon \;=\;1\;+\;i\sigma_{g}/\varepsilon_{0}\omega t_{g}$. The Femi energy *E*_f_ and electron mobility are 0.7 eV and 10,000 cm^2^/Vs for graphene. In this work, all transmission spectra and electric field distributions were calculated by the commercial software package “EastFDTD, version 5.0”, which is based on the well-known finite difference time domain method for the numerical simulation of the propagation of electromagnetic waves. For the details of the commercial software package, please visit the website (https://www.eastfdtd.com). In the *z* axis direction, two perfectly matched layers (PML) were set to avoid the scattering of electromagnetic waves at the boundary of the calculated region. In the *x* and *y* axis directions, two periodic boundary conditions were applied. A Gauss pulse was set as the light source, and the transmission spectra were obtained by Fourier transform. The electric field distributions on a plane can be recorded directly. In the regions of graphene and SRR, the minimum mesh size was set to be 0.05 and 5 nm. For the other region, the homogeneous mesh size was set to be Δ*s* = 20 nm, and the corresponding time step Δ*t* = Δ*s*/2*c*, where *c* is light speed in vacuum. Our designed hybrid structure can be fabricated by the following process: The gold SRR on the silica substrate is firstly fabricated by electron beam lithography. Then, a dielectric spacer with thickness of hundreds of nanometers is deposited on top of the array of the gold SRR structure by the electronic beam evaporation method. After that, a monolayer graphene is transferred onto the dielectric spacer. Finally, the GNRD is obtained by the second electron beam lithography or by the ultraviolet lithography with high precision alignment.

## 3. Results and Discussion

**Figure 2:**
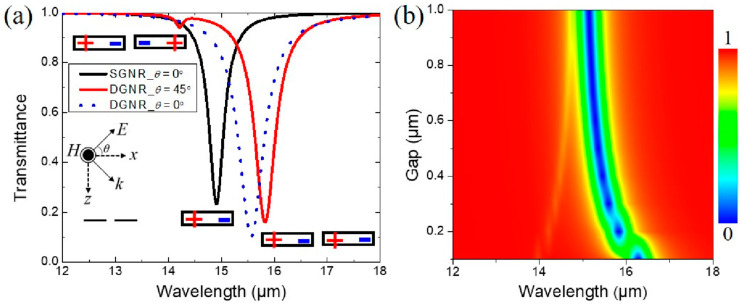
(**a**) Transmittance of the graphene nanoribbon dimer (GNRD) with *d*_1_ = 0.2 μm, with the inclined incidence angle (*θ*) being 0° and 45°, and the single GNR with *θ* being 0°. The insets of (**a**) show the corresponding plasmon mode for each spectral dip. (**b**) Transmittance of the GNRD under inclined incidence (*θ* = 45°) of light with various *d*_1_.

Beginning with the design of the dark elements, we numerically calculated the plasmon hybridization of the GNRD. The simulated transmittance spectra of a single GNR and the in-plane GNRD are shown in Figure 2a with an inclined incidence angle (*θ*) of 0° and 45°. Here, the width of the GNR and the gap size between the in-plane GNRD are 0.65 and 0.2 μm. For a normal incident TM wave, an electric dipole resonant mode of a single GNR is located at 15 μm, shown by the black solid curve in Figure 2a. As two GNRs are placed together with a small gap under inclined incidence (*θ* = 45°), two new plasmonic dipole bonding and antibonding [26,27] modes of the GNRD are generated at 14.2 and 15.8 μm through the near-field coupling, shown in the red solid curve in Figure 2a. The corresponding charge oscillations are also shown in the inset of Figure 2a. For comparison, the transmittance of the GNRD under normal incidence is also shown in the blue dashed curve in Figure 2a. Due to the net electric dipole moment of the antibonding mode being zero in the quasistatic limit, the antibonding mode of the GNRD is a real narrow dark mode, which could not be optically excited under normal incidence. The dark antibonding mode can only be excited weakly under inclined incidence owing to the retarded potentials. To the best of our knowledge, this is the first time researchers have discussed the effect of the graphene plasmonic dark mode. Figure 2b demonstrates that the splitting degree of the bonding mode and the antibonding mode is enlarged as the gap of the two GNRs decreases under inclined incidence, which is consistent with the theory of plasmon hybridization [38].

**Figure 3:**
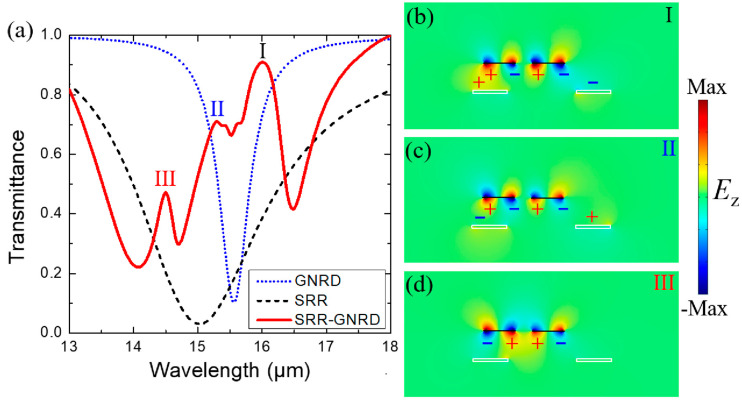
(**a**) Transmittance of the array of the hybrid graphene–metal metamaterial with the structural parameters *d*_1_, *d*_2_, and *d*_3_ = 0.4, 0.6, and 0.4 μm, respectively (red solid curve). The transmittance of the split-ring resonator (SRR) (black dashed curve) and GNRD (blue dotted curve) is also shown. (**b**–**d**) Simulated normalized electric field distributions of *E*_z_ and the surface charge distribution from the front view (in *xz* plane) for each plasmon mode (I, II, and III) shown in (**a**). I, II, and III represent the electric dipole in-phase bonding dark mode, out-of-phase bonding dark mode, and antibonding dark mode, respectively.

**Figure 4:**
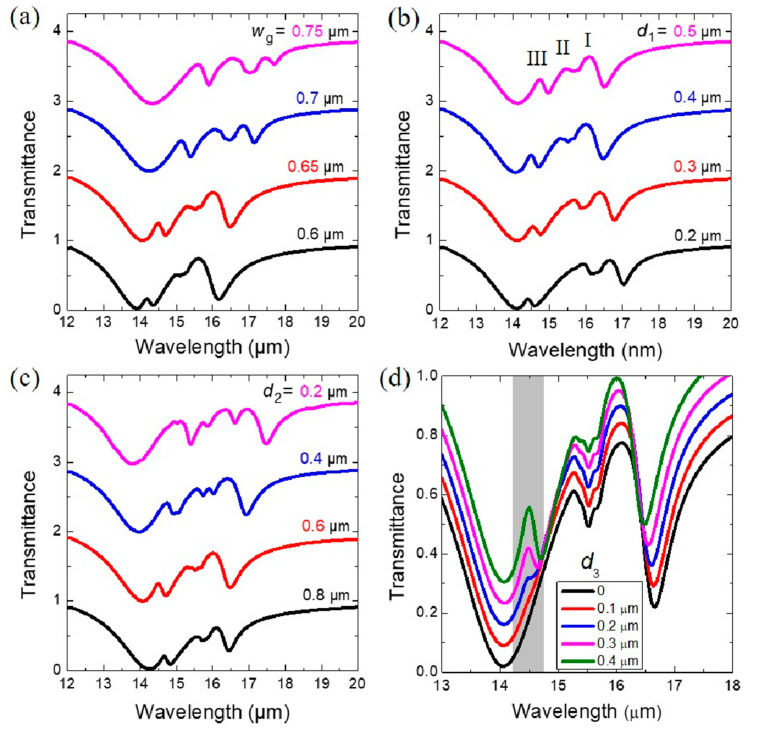
The normal-incidence transmittance of the hybrid structure with various (**a**) widths (*w*_g_) of the GNR, (**b**) gap sizes (*d*_1_) of the GNRD, (**c**) vertical distances (*d*_2_) between the GNRD and gold SRR, and (**d**) offsets (*d*_3_) between the geometrical centers of the GNRD and the gold SRR along the *x* axis.

Figure 3a presents the calculated normal-incidence transmission spectra of the optimized hybrid GNRD and gold SRR metamaterial. The structural parameters of the SRR and GNRD are set as same as those in Figure 1. The other parameters *d*_1_, *d*_2_, and *d*_3_ are set as 0.4, 0.6, and 0.4 μm, respectively. The Femi energy *E*_f_ and the graphene’s electron mobility are fixed at 0.7 eV and 10,000 cm^2^/Vs. The multiple narrow modes shown in the red curve in Figure 3a are obtained, which appear as three transparency windows (labeled I, II, and III) inside a broad resonant mode. The broad resonance is the fundamental magnetic resonance mode of the SRR, which corresponds to the transmittance of an individual gold SRR as shown in the black dashed curve. The two narrow peaks I and II correspond to the electric dipole bonding mode of an individual GNRD as shown in the blue dotted curve. The normalized electric and magnetic field distributions of both the fundamental magnetic resonance mode of an individual gold SRR and the electric dipole bonding mode of an individual GNRD are shown in Figure S1 in the supplemental material. The normalized electric field distributions of *E*_z_ and surface charge distribution from the front view (in *xz* plane) for the two narrow modes (I and II) in Figure 3b,c show that the electric dipole bonding mode of the GNRD further couples with the magnetic resonance mode of the gold SRR to generate the two plasmonic hybridized in-phase and out-of-phase dark modes. The third narrow peak III shown in Figure 3a represents the electric dipole antibonding mode with its *E*_z_ and surface charge distribution shown in Figure 3d. It is noteworthy that this electric dipole antibonding mode of the GNRD, as well as the Fano peak III under normal incidence, is only achieved by introducing structural symmetry breaking (*d*_3_ = 0.4 μm), which is further discussed in Figure 4d. The destructive interference between the triple narrow hybridized plasmon modes of the GNRD and the broad magnetic mode of the gold SRR leads to the generation of triple FRs in our hybrid metamaterial.

We further demonstrate the influence on the multiple FRs by varying the geometric parameters of the proposed hybrid structure. Figure 4a shows that the three Fano peaks redshift clearly as the width (*w*_g_) of the GNR increases. Figure 4b shows the transmittance of the hybrid structure with the gap size *d*_1_ between the GNRD being tuned from 0.2 to 0.5 μm. The resonant positions of the two Fano peaks (I and II) blueshift as *d*_1_ increases, while the resonant position of the Fano peak III slightly redshifts as *d*_1_ increases, which shows the same variation tendency observed in the pure GNRD structure shown in Figure 2b. The parametrical study by varying the vertical distance *d*_2_ between the GNRD and gold SRR is presented in Figure 4c. As *d*_2_ decreases from 0.8 to 0.6 μm, the three transparency windows become more prominent. More interestingly, when *d*_2_ further decreases to 0.4 μm, more transmission peaks emerge in the same structure due to the enhancement of the near-field coupling between the GNRD and the gold SRR. Such similar multiple FRs in hybrid metal–graphene structures have also been reported in a previously published paper [39]. We further investigate the effect of structural symmetry breaking by considering the offset (*d*_3_) between the geometrical centers of the GNRD and the gold SRR along the *x* axis. Figure 4d shows that as *d*_3_ increases from 0 to 0.4 μm, indicating the enhancement of the degree of the structural symmetry breaking, the Fano peak III emerges and gradually becomes clear at around 14.5 μm.

**Figure 5:**
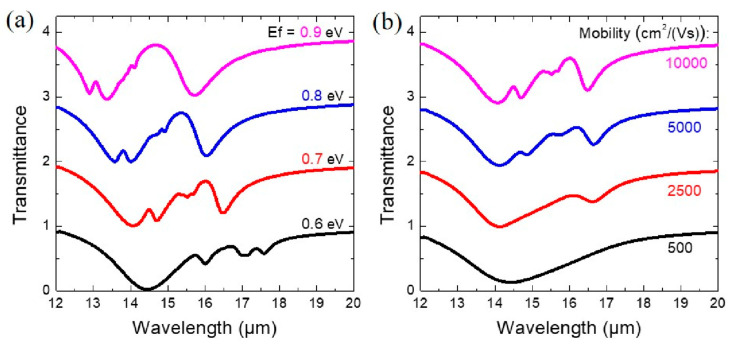
Transmittance of the hybrid structure with various (**a**) Fermi energies and (**b**) mobilities of the GNR.

The ability to actively control the optical properties of graphene by tuning its Fermi level is significant for dynamic optoelectronic devices. Figure 5a shows that the three Fano peaks obviously blueshift over a broad range as the Fermi level increases from 0.6 to 0.9 eV, while the structural parameters are unchanged. We further study the evolution of the optical property of the proposed hybrid metamaterial by decreasing the electron mobility (*μ*) from 10,000 to 500 cm^2^/Vs while keeping other parameters unchanged. Figure 5b shows that the three Fano peaks gradually become weaker as *μ* decreases from 10,000 to 5000 cm^2^/Vs. When *μ* further decreases to 2500 cm^2^/Vs, only one Fano peak with the corresponding narrow mode being the dipole bonding mode is observed. As *μ* becomes further smaller than 500 cm^2^/Vs, all three FRs disappear. This is due to the energy loss becoming dominant for the graphene with a small *μ*.

## 4. Conclusions

In summary, we have theoretically studied the tunable multiple Fano resonances in a hybrid metamaterial with decreased structural symmetry composed of an in-plane graphene nanoribbon dimer and gold split-ring resonator. The multiple Fano resonances are obtained by the near-field coupling between two hybridized narrow electric dipole bonding modes as well as a narrow dark electric antibonding mode of the graphene nanoribbon dimer and the broad fundamental magnetic resonance of the gold split-ring resonator. Interestingly, the Fano resonance with its corresponding dark mode of the antibonding modes in the graphene nanoribbon dimer is only excited by structural symmetry breaking. The multiple Fano modes can be tailored by controlling the structural parameters and Fermi levels. In addition, compared with the graphene nanopatch array, our designed hybrid nanostructure with a graphene nanoribbon dimer keeps continuous form with the benefit of preserving the high mobility of graphene and may simplify the possible fabrication processes. The dynamic multiple Fano resonances are achieved by varying the Fermi energy of graphene, which makes our proposed hybrid graphene–metal device more active than the metallic ones.

## Supplementary Materials

The following are available online at https://www.mdpi.com/2079-4991/10/12/2408/s1, Figure S1: Plasmonic property of individual gold SRR and individual graphene nanoribbon dimer. (a) Normalized electric field distribution and normalized magnetic field distribution from top view at fundamental magnetic resonance mode of gold split-ring resonators (*λ* = 15 μm). (b) Simulated normalized electric field distributions of *E*_z_ and surface charge distribution from front view (in *xz* plane) at the electric dipole bonding mode of graphene nanoribbon dimer (*λ* = 15.5 μm).
