# Supplementary material for: Graphene Multiple Fano Resonances Based on Asymmetric Hybrid Metamaterial
Source: Nanomaterials (Basel). 2020 Dec 2;10(12):2408. doi: 10.3390/nano10122408 (PMC7761262; doi:10.3390/nano10122408)
Supplement: Supplementary file 1 [file nanomaterials-10-02408-s001.pdf]

Supplemental material

Graphene Multiple Fano resonances based on asymmetric hybrid metamaterial

Zhendong Yan[1], Zhixing Zhang[1], Wei Du[2], Wenjuan Wu[1], Taoping Hu[1], Zi Yu[1], Ping Gu[3], Jing Chen[3], and Chaojun Tang[4,*]

[1] College of Science, Nanjing Forestry University, Nanjing 210037, China

[2] College of Physics Science and Technology, Yangzhou University, Yangzhou 225002, China

[3] College of Electronic and Optical Engineering & College of Microelectronics, Nanjing University of Posts and Telecommunications, Nanjing 210023, China

[4] College of Science, Zhejiang University of Technology, Hangzhou 310023, China

[*] Correspondence: chaojuntang@zjut.edu.cn

Contents

1. Plasmonic property of individual gold SRR and individual graphene nanoribbon dimer

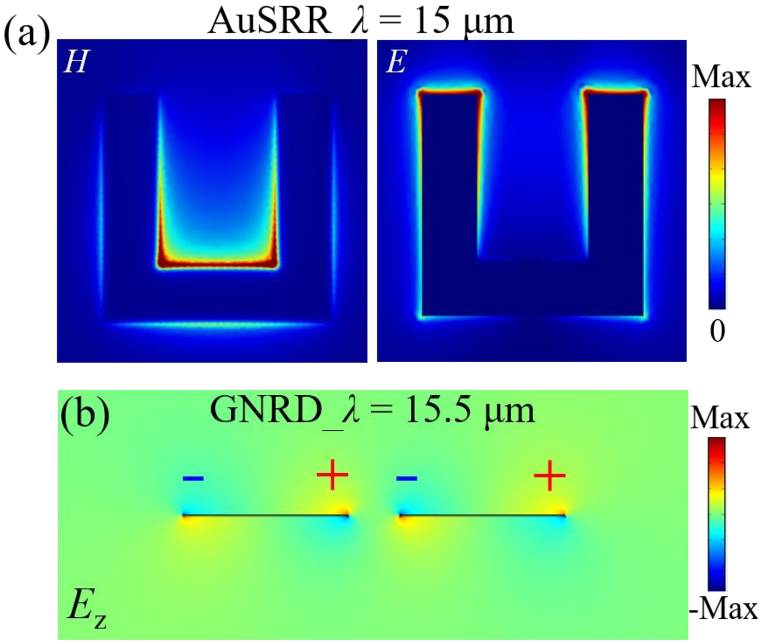

Figure S1. (a) Normalized electric field distribution and normalized magnetic field distribution from top view at fundamental magnetic resonance mode of gold split-ring resonators (λ = 15 μm). (b) Simulated normalized electric field distributions of E_z and surface charge distribution from front view (in *xz* plane) at the electric dipole bonding mode of graphene nanoribbon dimer (λ = 15.5 μm).
